# Estimating the risk of environmental contamination by forest users in African Swine Fever endemic areas

**DOI:** 10.1186/s13028-022-00636-z

**Published:** 2022-07-27

**Authors:** Vincenzo Gervasi, Andrea Marcon, Vittorio Guberti

**Affiliations:** Istituto Superiore per la Protezione e la Ricerca Ambientale, Via Ca’ Fornacetta, 9, 40064 Ozzano Emilia, BO Italy

**Keywords:** ASF, *Asfaviridae*, Bio-security, Epidemiology, Infected carcass, Pigs, *Sus scrofa*, Wild boar hunting

## Abstract

**Background:**

African Swine Fever (ASF) is a highly lethal viral disease caused by the African Swine Fever Virus (ASFV), the only virus of the *Asfaviridae* family, which affects different species of wild and domestic suids, and for which no vaccination or effective medical treatment is currently available. The virus can survive for long periods in the environment, and humans can unintentionally act as vectors through infected fomites, a risk that is linked to the ASF introduction into pig farms. We ran a simulation study, in which we reconstructed the probability process leading to the different forms of human-mediated ASF contamination in ASF endemic areas. We compared the infection risks related to different types of human forest activities and produced estimates of the minimum expected number of human-induced contamination events occurring annually at the scale of some European countries.

**Results:**

When analysed on a short temporal scale and in a relatively small spatial context, ASF environmental contamination appeared as a rather unlikely event for most of the simulated forest uses, with contamination probabilities often lower than 0.1%. When scaling up the contamination process to a whole year and to large geographic areas, though, the accumulation of the same forest activities, repeated several times per month within the same patch of forest, produced the expectation that thousands of contamination events would occur each year, with potentially relevant epidemiological consequences. Wild boar supplemental feeding and forest logging emerged as the riskiest activities in terms of contamination probabilities, but risk was highly influenced by the frequency and intensity of the different types of forest use.

**Conclusions:**

The risk of human-mediated ASF environmental contamination should not be disregarded when planning management actions to reduce ASF circulation and prevent its breach into the pig farming system. Supplemental feeding should be strongly reduced or avoided in ASF affected areas. Wild boar hunting, which is often employed as an active management tool in ASF affected areas, should be seen as both a tool for controlling wild boar density and as a potential risk for further contamination. It is essential to implement and enforce strict biosecurity measures for all forest-based human activities in ASF endemic areas.

**Supplementary Information:**

The online version contains supplementary material available at 10.1186/s13028-022-00636-z.

## Background

African Swine Fever (ASF) is a highly lethal viral disease, caused by a virus of the *Asfaviridae* family, which affects different species of wild and domestic suids, and for which no vaccination or effective medical treatment is currently available [1]. During the last 15 years, and after its accidental human-caused introduction to Georgia, the infection has progressively affected most of the Eastern European and South-East Asian countries, causing a drastic reduction in wild boar (*Sus scrofa)* and wild pig densities in the affected areas, before entering an endemic state at low wild boar densities and low virus prevalence [2, 3]. Having also breached into the intensive food production system of pig farming, ASF is currently both an ecological and an economic concern, which causes large direct and indirect economic losses to the pig industry [4].

It is now well established that the main epidemiological mechanism that allows ASF to persist is not related to the direct contact between live infected individuals and susceptible animals, but it is rather due to the function of infected wild boar carcasses and other forms of environmental contamination acting as virus reservoirs [5, 6]. During recent years, the main research focus has been understandably dedicated to exploring the role of infected carcasses in virus transmission and of wild boar behaviour towards carcasses as a driver of infection risk [7]. Carcasses, though, are not the only matrix in which the ASF virus can survive for long periods: in sandy soils, the virus can persist for several weeks after a carcass has been removed [8]; urine and faeces have been experimentally shown to act as a potential infection source for 3–15 days after deposition, depending on season and temperature [9]; wild boar offal, resulting from wild boar dressing after hunting, can be infective for as long as the whole carcass, depending on the environmental temperature [10]. Moreover, although wild boar and other scavenging species have been shown to act as potential vectors of ASF contamination and spread [11], they are not the only actors playing this role in the ASF endemic areas of a human-dominated landscape such as Europe.

Humans, too, visit and use forests both for recreational and professional reasons. Hiking, mushroom and berry picking, hunting, and wood logging are examples of intensive human presence in ASF affected areas. Some of these forest activities, such as wild boar hunting or wild boar supplemental feeding, are directly oriented towards the species virus reservoir in the whole of Eurasia, thus creating a spatial proximity between many forest users and the environmental sources of ASF contamination (urine, droppings, blood, etc.). As the ASF virus can contaminate fomites, such as clothes, footwear, and equipment, and persist for long times on their surface [12], the potential for human forest users to accidentally step on or touch infected material and to act as an ASF vector should be considered.

Accordingly, most of the negative economic consequences of ASF on pig farming and meat production occur at the interface between forest and farm, with humans often playing a major role both in the long-distance virus dispersal and in the ASF intrusion into pig holdings [13]. On several occasions, such as in the Czech Republic, Poland, Hungary, and Belgium, the indirect human-mediated long-distance spread of ASF virus initiated new isolated clusters of infection in wild boar, some of which developed into long-lasting outbreaks [14]. Moreover, a plethora of empirical studies have shown that the main risk factors, which can promote the introduction and spread of the virus at the farm level, are related to poor farming practices and, more generally, to low biosecurity levels at farm levels [15–17]. Although the risks associated with human-mediated environmental contamination have been highlighted as an additional component of the complex ASF infection routes [14], their likelihood and potential epidemiological impact have not been quantified so far, also due to the complexity and unpredictability of the whole risk process, which involves a series of highly unlikely events (such as stepping on infected wild boar droppings or touching an infected portion of forest soil) over very large areas and for relatively long periods of time.

Here, we present the results of a simulation study, in which we tried to mechanistically reconstruct the probability process leading to different forms of human-mediated environmental ASF contamination in ASF endemic areas. We compared the infection risks related to different types of human forest activities with a particular focus on those activities, such as hunting and supplemental feeding, which generate a spatial overlapping correlation between wild boar and human movements in the forest. Supplemental feeding, in particular, has been highlighted as a factor potentially increasing the risk of ASF spread, both for its effect on wild boar density and for its potential to create large numbers of individuals around the same spot [18]. We also assessed how the environmental contamination risk varied as a function of the different seasonal and ecological conditions, and produced realistic estimates of the minimum expected number of human-induced contamination events occurring annually at the scale of all European countries. Our focus was on wild boar infected droppings as potential sources of environmental contamination. We discuss our results in the context of the efforts to reduce and control both the geographic spread of ASF and its negative consequences on pig farming.

## Methods

### Model structure

To assess the risk that the ASF virus could be carried by forest users in an infected ASF endemic area, we built a series of mechanistic, spatially-explicit Monte Carlo simulation models in R 4.1.2 [19], which mimicked the different types of forest use by humans in an ASF endemic area. All model details, including R scripts, are available in the Additional files 3, 4, 5, 6. We modelled the following processes that could potentially affect the probability of environmental contamination:

1. The spatial distribution of wild boars in the study area;
2. The disease prevalence and the resulting distribution of infected individuals;
3. The spatial distribution of infected wild boar droppings, resulting from the equilibrium between dropping production and decay;
4. The trajectories of forest users and their probability of stepping on an infected dropping, thus carrying the virus away from the study area.

To build the simulated environment, we defined a 50 km^2^ study area, in which we simulated two different wild boar populations of 50 and 150 individuals, respectively, corresponding to a population density of one and three wild boars/km^2^. To distribute all wild boars in the simulated environment, we randomly selected individual home range centres in the study area, and built a circular buffer around it, to identify their home range area. We initially set home range size to 1 km^2^, based on wild boar spatial ecology [20]. Then, we fixed the ASF prevalence in the area to 2%, which is consistent with that reported in several Eastern European countries after the end of the first ASF epidemic breakout [21, 22].

The following step of the simulation process was to identify the ASF infected wild boars within the population. We randomly selected 2% of the home range centres and reduced their home range area to half to account for the reduced movement capabilities of ASF infected wild boars during the acute phase, considering that most infected animals are likely to die within 5–10 days from the initial infection [23]. In order to simulate the number and spatial distribution of infected wild boar droppings present within each home range at any moment, we accounted for the temporal equilibrium between wild boar defecation rates and the decay of the ASF virus in the droppings with time. We derived daily defecation rates (DDR) from Ferretti et al. [24], who estimated them to be on average 3.8 droppings per day during summer and 4.3 droppings per day during winter. Persistence times of the ASF virus in droppings, and more generally in the environment, have been shown to be highly variable depending on temperature and humidity [3]. We defined an average virus persistence in wild boar droppings of 5.1 days in summer and 8.5 days in winter, based on the results obtained on experimentally infected pigs in a captive environment [9]. The overall number of infected droppings in the simulated study area at any given moment, calculated as the product between population size, virus prevalence, defecation rate and virus persistence, was 20 during summer and 36 during winter. Given such large seasonal differences in the presence of infected droppings, we performed two separate sets of simulations, one with winter and one with summer parameters, in order to assess the seasonal variation in the risk of environmental contamination.

### Types of forest use and associated trajectories

After generating a simulated environment, a wild boar population, and an ASF endemic condition, we set up a series of simulated movement algorithms, to compare the potential for five different types of forest use to act as vectors for an anthropogenic ASF virus spread. To this aim, we considered the following five forest-based activities:

1. Movement of single forest users, such as mushroom/berry pickers, hikers, runners, etc.;
2. Collective wild boar hunting, performed by a group of hunters moving in parallel lines in a pre-defined hunting ground;
3. Individual wild boar hunting, performed by a single hunter, driven by dogs towards the home range of the closest wild boar;
4. Wood logging, performed by a group of forest workers in a relatively small, but intensively used forest patch;
5. Periodic visits to a wild boar feeding site, for food replenishment.

Each of the five types of forest use differed in terms of the length, spatial arrangement, and movement rules generating the resulting walking trajectories. To simulate the trajectory of a single person walking through the infected area, under the risk of stepping on an infected wild boar dropping, we generated a random walk process with a total length of 10 km, divided into 10 1-km segments. We initially generated a starting point at random in the study area. Then, we randomly selected an initial moving direction by generating a random angle. At the end of each segment, we generated a new turning angle to define the new direction of the trajectory and removed those angles which led the trajectory outside the study area, thus creating a bouncing border.

For the scenario simulating a wild boar collective hunt, we divided the study area into 50 1-km^2^ hunting grounds. Then, we randomly selected one of them, in which we generated 30 parallel trajectories at about 33 m distance from each other, which crossed the hunting ground twice, for a total length of 2 km for each individual trajectory. In the third scenario, we mimicked the movement of a single hunter walking through the forest with a pack of hounds, thus actively searching for wild boars and pushing them towards a shooting front. To this aim, we used the same mosaic of 1-km^2^ hunting grounds used for the previous scenario. Then, if the hunting ground overlapped (even partially) with a wild boar home range, we defined the centre of that home range as an attraction point for the hunter’s movement and used an Ornstein–Uhlenbeck function [9] to generate a 5-km trajectory that accounted for such dog-derived attraction force. We simulated both hunting scenarios only in winter to account for the most common annual distribution of wild boar hunting periods.

**Fig. 1:**
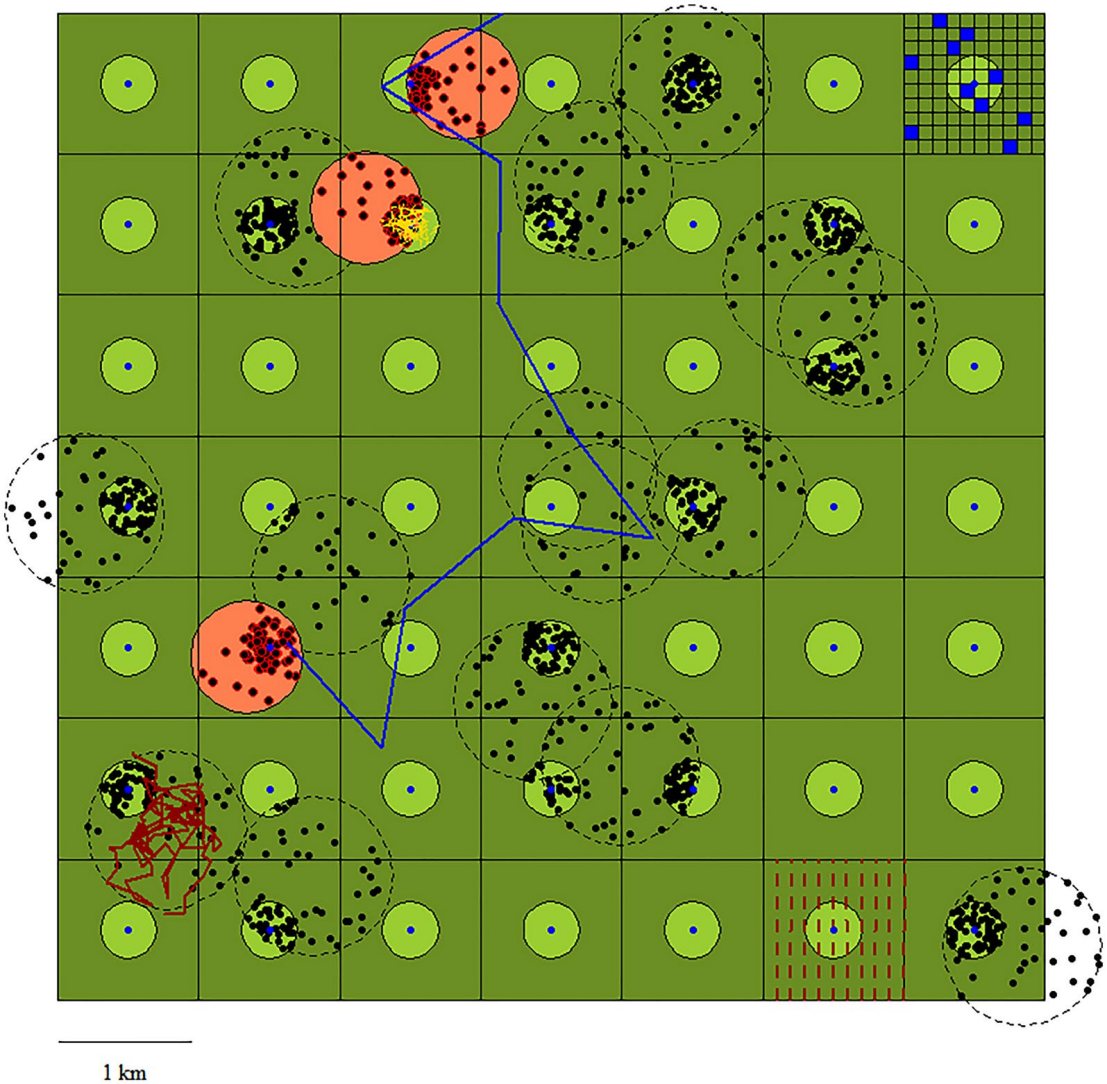
Spatial arrangement of the elements used to simulate the risk of ASF environmental contamination by five types of forest users. Total study area = 50 km^2^; large squares = 1 km^2^ plots, used to simulate wild boar hunting; small blue squares = 3-ha plots used to simulate wood logging activities; light green circles = 10-ha plots identifying wild boar supplemental feeding stations (blue dots in the centre); dashed circles = simulated home ranges of ASF-free wild boar; red circles = simulated home ranges of ASF infected wild boar; black dots = wild boar droppings (infected droppings are circled in red); blue line = trajectory of an individual user; red lines = hunters’ trajectories, both collective (straight lines) and individual (broken line); yellow line = walking trajectory during a visit at a feeding station. The figure is a graphical representation of all the elements included in the simulations, although only some of them were included in the calculations in each scenario

To simulate the activity of forest loggers, we divided the study area into 1667 3-ha parcels, then randomly selected one of them, which was used to simulate wood logging. In the selected parcel, we simulated that three people would work for a period of 5 days, randomly walking 1 km each day within the forest parcel. Finally, to simulate a visit to a wild boar feeding site, we randomly generated a 10-ha plot in the study area, with a feeding site in the centre. Then, if the feeding site overlapped with a wild boar home range, we forced dropping density within 100 m from the feeding point to be double than further away, thus mimicking the intensive use of feeding sites by wild boar. We simulated that a single visitor would walk one km within the feeding area, and used the Ornstein–Uhlenbeck function described above, to attract the trajectory to the feeding site. As the practice of supplemental feeding to wild boar has the potential to affect wild boar spatial behaviour, creating a more clustered spatial distribution of droppings than in areas in which feeding is not used as a management tool, we considered the effect of such practice not only on the visit at the feeding site itself, but also on the other four types of forest use. To account for this additional risk, we defined and ran a second set of simulations, in which we mimicked the existence of a network of supplemental feeding stations in the study area at a density of one feeding station/km^2^, in line with the observed spatial distribution of feeding stations in Europe [25, 26]. Then, for each type of forest use, we compared the contamination risk with and without the clustering effect of supplemental feeding. The overall spatial arrangement of all the elements included in the simulation study are shown in Fig. 1.

### Estimation of contamination probabilities

After generating the trajectories for each type of forest use, we estimated the resulting associated risk of ASF environmental contamination. We measured the linear distance between each ASF infected wild boar dropping and each trajectory. We retained only those droppings whose distance from the trajectory was shorter than 20 cm. As a human walking trajectory is made of actual steps (when the foot touches the ground) and of strides (when the foot moves forward without touching the ground), we estimated that only about 1/3 of the trajectory was at risk of stepping on a dropping. To account for this part of the process, we extracted a random number from a binomial distribution, with the number of trials equal to the number of droppings on the trajectory, and a success probability equal to 0.33. This provided us with the effective estimated number of infected droppings on which a person would step while moving on the simulated trajectory, assuming that each time a user stepped on an infected dropping, the virus would remain attached and would be carried away. As the absolute likelihood of a contamination event was expected to be rather low, we replicated the model over 100,000 iterations for each simulated scenario. The proportion of model runs in which a forest user stepped on a infected dropping provided the probability for an ASF contamination to occur under a set of simulated conditions.

After running all model scenarios (winter vs. summer, high density vs. low density, with and without supplemental feeding) for all the simulated types of forest use, we compared the resulting contamination probabilities. Initially, we compared contamination risks associated with each type of forest use, using the intrinsic characteristics of their specific movement patterns as a metric, irrespective of their different spatial and temporal distribution, which were determined by how often a certain activity was carried out, the size of the area used, or by how many people were involved, etc. To this aim, we extracted the contamination probability resulting from a 1-km trajectory of a single person for each type of forest use in each simulated scenario. This first level model evaluation provided us with an initial estimate of how intrinsically risky a certain movement pattern was in terms of ASF contamination.

### Monthly contamination rates

After estimating the contamination risk associated with a single event of forest use, we scaled up the risk assessment process, by estimating the expected number of monthly and yearly contamination events associated with a certain spatial and temporal pattern, which resulted from the frequency and intensity of a given type of forest use. For the individual use (hiker, mushroom picker, etc.), we considered a local population density of 10 persons/km^2^, which corresponds to the population density in the rural areas of countries, such as Estonia and Latvia, in which ASF is becoming endemic (data.worldbank.org). Then, we assumed that 20% of the local population would use the forest once a month, thus resulting in a total of 100 individual forest users visiting the 50 km^2^ forest each month. Starting from this baseline scenario, we ran the simulation model for a period of 30 days, during which we simulated 100 10-km random trajectories.

For the scenario of wild boar collective hunting, we assumed that all 50 hunting grounds, each 1 km^2^ in size, would be used once a month during the hunting season. Similarly, we assumed that each hunting ground would also be used once a month for an individual hunting trip with dogs. For the forest logging scenario, we simulated that 10% of the overall forest surface would be cut, resulting in a total of 14 3-ha plots used each month. Finally, we simulated a density of one supplemental feeding station/km^2^, and assumed that each feeding station would be visited by one person each month. After running all these additional model scenarios, we estimated the monthly contamination probability associated with each scenario, in the same way as described above. The characteristics of each type of forest use and the frequency/intensity of use are summarized in Table 1.

**Table 1:** Summarized description of the simulated scenarios used to estimate the probability of environmental contamination in African Swine Fever endemic areas

Type of forest use	Plot area (km^2^)	Total no. plots considered	No. plots used in a month	No. visits per month	No. persons/visit	km/person/visit	Type of walk
Individual (hiker, mushroom/berry picker, etc.)	50	1	1	100^a^	1	5	Random
Wild boar hunt (collective drive)	1.0	50	50	1	30	2	Linear
Wild boar hunt (single hunter with dogs)	1.0	50	50	1	1	5	Attraction point
Forest logging	0.03	166	14^b^	5	3	1	Random
Feeding site visit	0.1	50	50	1	1	1	Attraction point

Each scenario was run in a simulated 50 km^2^ study area over 100,000 iterations

^a^The number of visits per month corresponds to a rural area with a human density of 10 individuals/km^2^, in which 20% of the population visits the forest once a month

^T^he number of plots used each month for forest logging corresponds to an area in which 10% of the total forest surface is logged each year

### Sensitivity analysis

As the definition of modelling scenarios involved a series of arbitrary decisions about model parameters, we also performed a sensitivity analysis to assess how the contamination risk was sensitive to changes in these subjective parameter values. To this aim, we re-ran the model in a range of increasing individual forest users, ranging from 0 to 5000; in a range of increasing frequency of wild boar hunting trips, ranging from 0 to 10 per month in a single hunting ground; in a range of increasing percentage of forest logged each year, ranging from 0 to 100%; in a range of increasing density of supplemental feeding stations, ranging from 0 to 3/km^2^. The sensitivity analysis also allowed us to produce contamination risk estimates for a range of different field conditions.

### Yearly and country-level expected contamination events

After estimating monthly contamination probabilities, we also scaled them up to predict the total number of contamination events expected to occur in the 50 km^2^ study area in a year. To calculate expected contaminations, we extracted 1000 random numbers from two binomial distributions, one for summer and one for winter contamination risk, in which the number of trials corresponded to the number of months in each season (6 for spring/summer, 6 for fall/winter), while success rate was given by the estimated monthly contamination risk of each type of forest use. The resulting frequency distribution of the total number of predicted contamination events allowed us to calculate an average estimate and the associated 95% confidence intervals.

Finally, as an exercise to provide realism to our modelling effort, we tried to further scale up the estimates of ASF contamination rates, producing an expected total number of ASF contamination events at the country level, for five eastern European countries in which ASF is entering into an endemic state, namely Estonia, Latvia, Lithuania, Poland, and Romania [27]. The estimation had no ambition to be especially accurate, given the many unaccounted confounding factors and the simple type of calculations adopted. We had the aim to provide an order of magnitude of the number of potential ASF contamination events that the whole set of human activities taking place in the forest could generate. For each of these countries, we collected information about the total surface, total human population density, proportion of land covered by forest, and human population density in rural areas (data.worldbank.org). Then, we extrapolated the number of contamination events derived from the 50 km^2^ area to the total forest surface of each country, adjusting the expected number of individual forest users for the different country-specific human density values, while assuming that hunting and forest logging practices would not be different among countries. We estimated total contamination events both with and without wild boar supplemental feeding.

## Results

### Single-use contamination probabilities

**Fig. 2:**
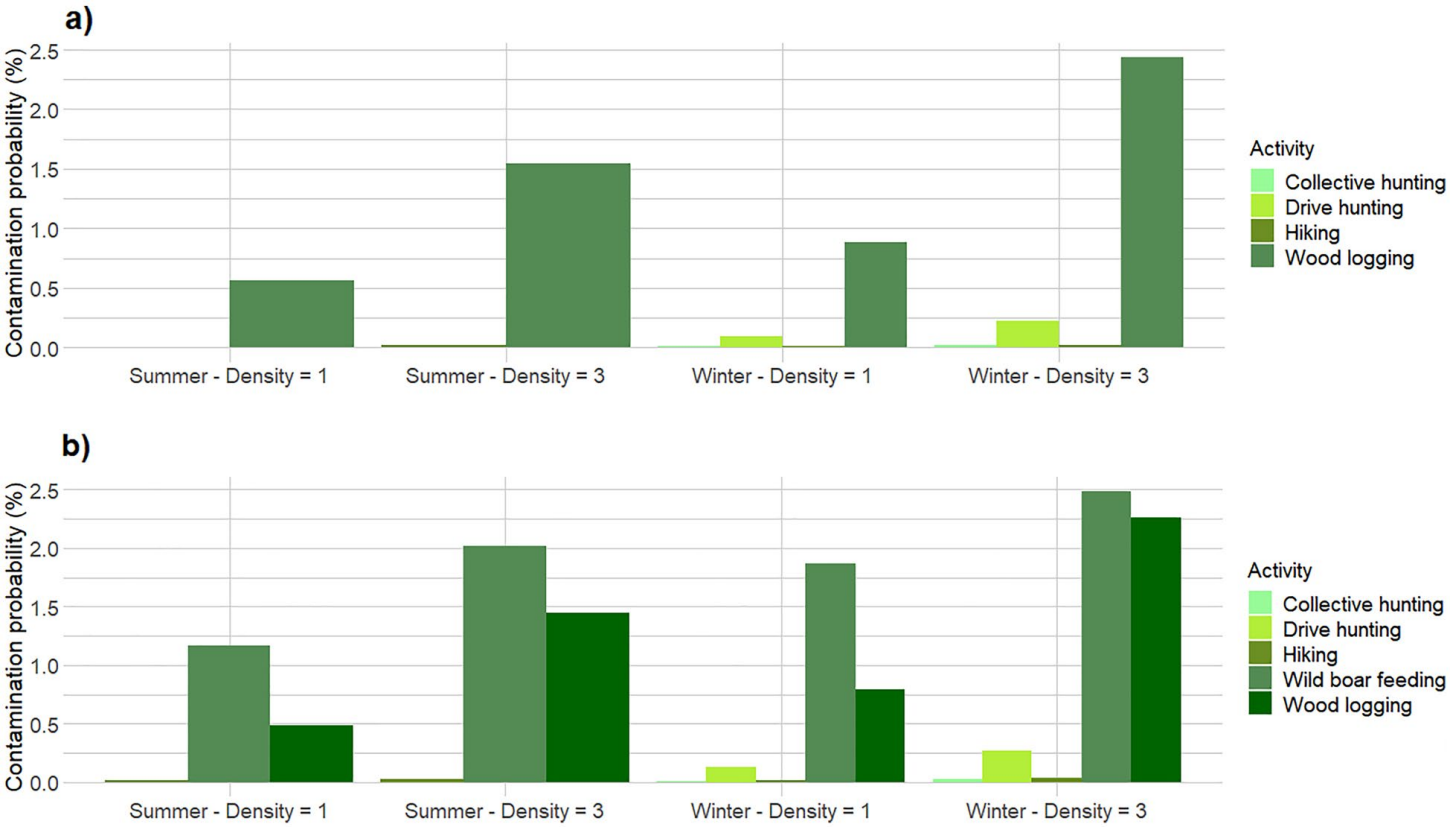
Probability of African Swine Fever environmental contamination, corresponding to a 1-km simulated walk in a 50 km^2^ forest in which ASF is endemic (prevalence = 2%). The contamination probabilities are expressed in percentage and are provided for five types of forest use, at different wild boar densities and in different seasons, without (**a**) and with (**b**) wild boar artificial feeding

When comparing the intrinsic risk of ASF environmental contamination among the different types of forest use, estimated on a single 1-km trajectory, the visit to a wild boar feeding site emerged as the riskiest activity, followed by forest logging. A single km walked around a supplemental feeding site exhibited a contamination probability ranging from 1.17 to 2.49%, depending on wild boar density and on the season (Fig. 2b). These estimates corresponded to one contamination event for every 41–87 km walked around a feeding station. As expected, contamination risk increased with higher wild boar density, and in winter than in summer (Fig. 2a, b). In comparison, one km walked inside a forest logging parcel corresponded to a contamination probability ranging from 0.48 to 2.26%, with one expected contamination event occurring on average every 44–209 km walked. The other three simulated types of forest activity exhibited much lower intrinsic contamination probabilities, when estimated on a single km (Fig. 2a, b). Between the two types of wild boar hunting, the individual, dog-driven hunt exhibited a higher intrinsic contamination risk than the collective hunt, with the first type producing on average a contamination event for every 376–1110 km walked, whereas the second produced on average a contamination event for every 3758–10,856 km walked. Finally, the individual forest use retained a similar intrinsic contamination risk as the one associated with collective wild boar hunting, generating on average a contamination event for every 3267–9695 km walked. The estimated contamination probabilities were higher in all the scenarios including wild boar supplemental feeding than in the ones in which this practice was excluded, except for forest logging, in which wild boar feeding caused a slight reduction in the estimated contamination rates (Fig. 2a, b). All the ASF contamination probabilities associated with a single 1-km trajectory are shown in Additional file 1.

### Monthly contamination rates

**Fig. 3:**
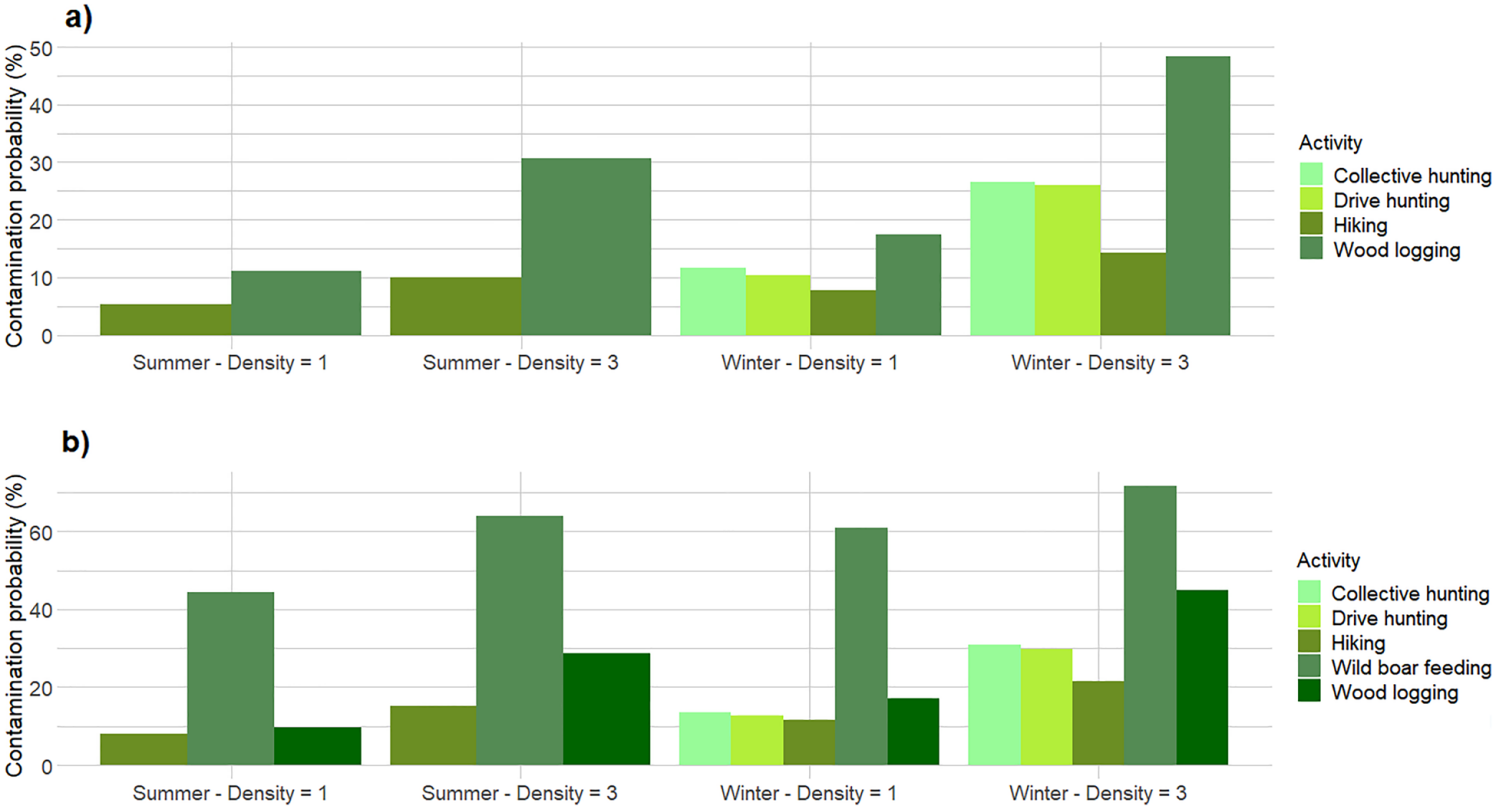
Probability of African Swine Fever environmental contamination, corresponding to five different types of forest use, simulated over a 30-day period in a 50 km^2^ forest in which ASF is endemic (prevalence = 2%). The contamination probabilities are expressed in percentage and are provided at different wild boar densities and in different seasons, without (**a**) and with (**b**) wild boar artificial feeding

When comparing the different types of forest use not only based on their intrinsic risk of environmental contamination, but more realistically based on their different spatial and temporal patterns (plot area, frequency of use, number of persons involved, etc.), wild boar feeding stations were still the hotspots of ASF contamination risk, with monthly contamination probabilities ranging from 44.4 to 71.6%, depending on the season and on wild boar density (Fig. 3b). This corresponded to an average of one contamination event occurring at feeding stations every 1.4–2.2 months. Forest logging was again the second most risky activity in terms of contamination risk (Fig. 3a, b), with one contamination event occurring on average every 2.0–10.6 months. Differently from what was assessed based on the single 1-km trajectory comparison, the two types of wild boar hunting techniques exhibited similar monthly contamination probabilities when compared on a monthly basis and accounting for the different number of hunters involved (Fig. 3a, b). Wild boar hunting was associated, on average, with one contamination event every 3.2–9.7 months. Finally, also in this case the individual forest users caused the lowest ASF environmental contamination risk (Fig. 3a, b). At the frequency of 100 users per month, the model predicted an average of one contamination event every 4.7–19.0 months. All the monthly ASF contamination probabilities are shown in Additional file 2.

### Sensitivity analysis

**Fig. 4:**
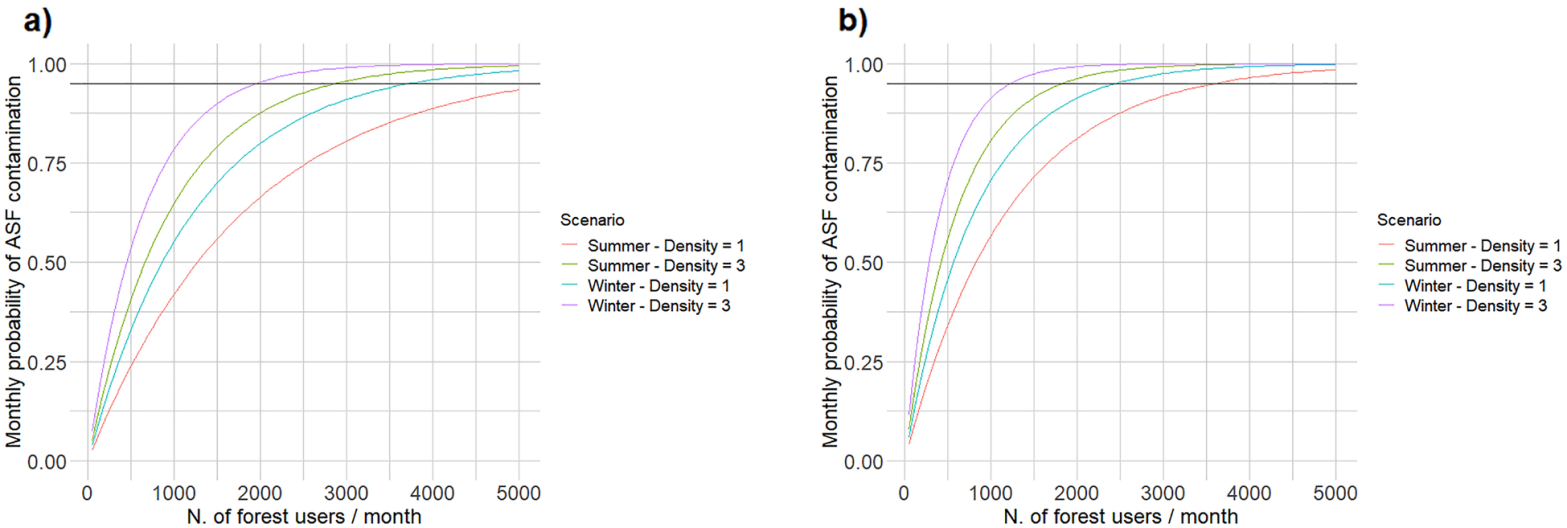
Sensitivity analysis of African Swine Fever environmental contamination risk to the number of monthly individual users (hikers, tourists, mushroom/berry pickers, etc.) visiting an ASF endemic area (area = 5 km^2^–ASF prevalence = 2%). The variation in contamination risk as a function of forest intensity of use is provided for areas without (**a**) and with (**b**) wild boar artificial feeding, for different seasons and at different wild boar densities

**Fig. 5:**
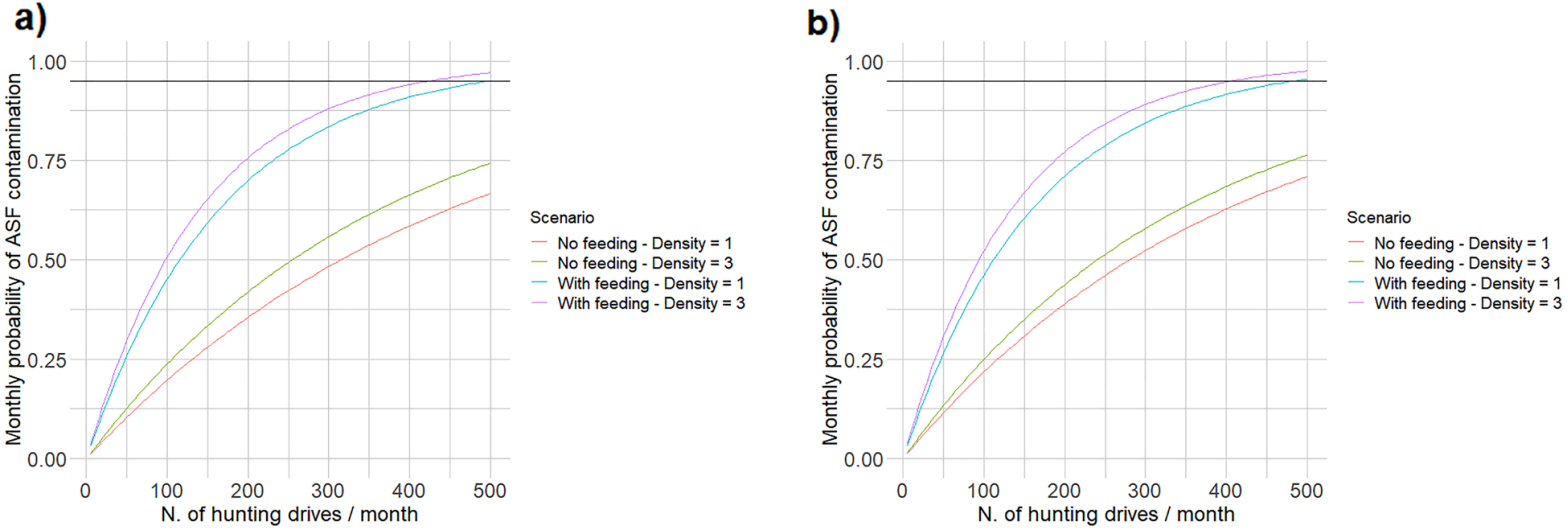
Sensitivity analysis of African Swine Fever environmental contamination risk to the number of monthly wild boar hunting trips occurring in an ASF endemic area (area = 50 km^2^–ASF prevalence = 2%). The variation in contamination risk as a function of hunting intensity is provided both collective (**a**) and individual (**b**) wild boar hunting, for different seasons and at different wild boar densities

**Fig. 6:**
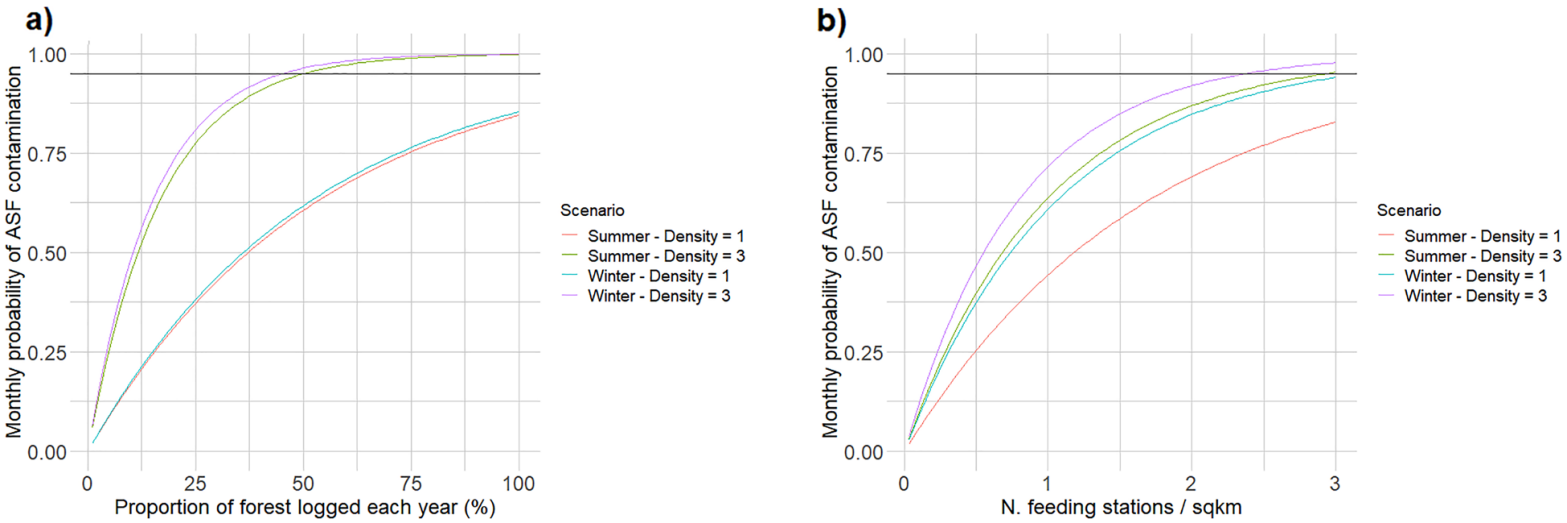
Sensitivity analysis of African Swine Fever environmental contamination risk to the proportion of forest logged each year (**a**) and to the density of wild boar feeding stations in an ASF endemic area (area = 50 km^2^–ASF prevalence = 2%). The variation in contamination risk as a function of forest logging and feeding intensity is provided for different seasons and at different wild boar densities

As expected, the estimated ASF contamination probabilities associated with the different types of forest use were sensitive to the choice of the specific parameters defining their intensity/frequency. For the individual, recreational forest use, the estimated contamination risk was rather low at the simulated intensity of use (100 users/month), but it rapidly increased when the number of monthly visitors increased: at high wild boar density (3/km^2^) in winter, 2000 visitors/month corresponded to a 95% probability of contamination, whereas 5000 visitors/month were necessary to produce the same contamination risk in summer at low wild boar density (1/km^2^; Fig. 4a, b). Similarly, both hunting techniques exhibited increasing contamination probabilities for an increasing number of hunting trips during the month (Fig. 5a, b). Contamination risk approached 95% when the total number of simulated hunting trips approached 500, which corresponded to about 10 hunting days in each hunting plot each month (Fig. 5a, b). For forest logging, contamination probabilities were very different between winter and summer (Fig. 6a), and in both cases were directly proportional to the proportion of forest surface logged each year. In particular, the likelihood of a contamination event approached 95% when about 50% of the forest was affected by some logging activity each year (Fig. 6a). Finally, the density of wild boar feeding stations was positively correlated with the associated risk of ASF environmental contamination. While contamination probabilities were rather high already at the simulated density of one station/km^2^, they approached 95% when the density was increased to about 2.5–3 stations/km^2^ (Fig. 6b).

### Yearly and country-level contamination rates

Resulting from the contribution of all the simulated types of forest use, in the different combinations of wild boar density and in the different seasons, our model estimated that in a forest area of 50 km^2^, an average of 3.8–9.2 contamination events would occur every year if no supplemental feeding was provided to wild boar (Table 2), whereas the expected number of yearly contaminations increased to 10.6–18.3 in areas with supplemental feeding (Table 2). Of these contamination events, about 54% were due to the periodic visits to wild boar feeding sites, 22% were due to forest logging activities, 18% was related to wild boar hunting (shared in similar portions between collective and individual hunting; Table 2), whereas about 7% of the contamination events were due to individual forest users (Table 2).

**Table 2:** Predicted number of yearly environmental contamination events occurring in an African Swine Fever endemic area (area = 50 km^2^)

Type of forest use	Wild boar density			
	1.0/km^2^		3.0/km^2^	
	Supplemental feeding			
	No	Yes	No	Yes
Individual	0.78 (0.70–0.85)	1.15 (1.08–1.25)	1.44 (1.34–1.54)	2.19 (2.08–2.30)
Wild boar hunt (collective drive)	0.70 (0.64–0.74)	0.80 (0.75–0.85)	1.59 (1.52–1.67)	1.84 (1.78–1.92)
Wild boar hunt (single hunter with dogs)	0.62 (0.58–0.67)	0.76 (0.71–0.82)	1.55 (1.48–1.62)	1.78 (1.71–1.85)
Forest logging	1.71 (1.60–1.81)	1.59 (1.50–1.68)	4.66 (4.59–4.88)	4.41 (4.26–4.54)
Feeding site visit	–	6.31 (6.17–6.45)	–	8.11 (7.97–8.25)
Total	3.81 (3.52–4.07)	10.61 (10.21–11.05)	9.24 (8.93–9.71)	18.33 (17.80–18.86)

The expected number of contamination events was derived from a spatially-explicit simulation model, and is provided for different types of forest use, at different wild boar densities and with/without wild boar artificial feeding. Numbers in parentheses indicate the 95% confidence intervals around the mean estimates

The extrapolation of our model estimates at a country level produced rather different predictions about the expected number of ASF environmental contaminations in the five countries considered, depending on the inclusion or exclusion of wild boar supplemental feeding. In absence of feeding, the expected number of contaminations ranged from a minimum of about 1900 per year in Estonia, to a maximum of about 7400 in Poland, mainly because of the high human density in rural areas in this latter country (Table 3). When considering the existence of wild boar supplemental feeding, the expected number of ASF contamination events was about three times higher, ranging from about 4,800 per year in Lithuania to more than 20,000 per year in Poland (Table 3).

**Table 3:** Extrapolation of the expected number of yearly African Swine Fever (ASF) environmental contamination events in 5 European countries in which ASF is endemic

Country	Country area (km^2^)	Forest area (%)	Population density (people/m^2^)	Population density in rural areas (people/km^2^)	Expected no. of ASF contaminations per year	
					No supplemental feeding	With supplemental feeding
Estonia	45,340	56.0	30.6	10.2	1938	5397
Latvia	64,570	54.9	30.6	10.0	2703	7527
Lithuania	65,290	35.1	44.6	15.0	1748	4869
Poland	312,690	30.9	124.0	54.6	7380	20,551
Romania	238,400	30.1	83.8	40.3	5471	15,235

Extrapolations were derived from the results of a spatially-explicit simulation model based on a 50 km^2^ study area, and provided for the scenarios with and without wild boar artificial feeding

## Discussion

The combination of our ASF contamination risk estimates, at different spatial and temporal scales, underlines the dual nature of the risk process: (i) when analysed on a short temporal scale and in a relatively small spatial context, ASF environmental contamination appeared as a rather unlikely event for most of the simulated forest uses (see Fig. 2a, b), with contamination probabilities often lower than 0.1%; stepping by chance on an ASF infected wild boar dropping when walking in a large patch of forest, is by definition an infrequent event; (ii) when scaling up the contamination process to a whole year and to large geographic areas, such as the ones currently affected by African Swine Fever, the accumulation of the same forest activities, repeated several times per month within the same patch of forest, and recurring with the same patterns over the whole wild boar geographic range in the European continent, produced the expectation that thousands of contamination events would occur each year (Table 3), with potentially relevant epidemiological consequences. Moreover, it should be noted that the number of contamination events predicted by our model in the different scenarios is only related to infected wild boar droppings. We know that droppings are only one among the several potential sources of ASF environmental contamination [8–10], and we should therefore consider our model predictions as a minimum estimate of the potential number of contamination events likely occurring in an ASF endemic area. On the other hand, our simulation study considered only one side of the ASF contamination process i.e., the probability that the ASF virus would be carried away from the forest by a human user. For the contamination to produce epidemiological consequences (in the wild or in a farm), the infected material needs to get in contact with a susceptible animal within the time frame during which the ASF virus can survive on the fomite. This means that only a portion (unknown and not quantified) of the contamination events predicted by our model will generate new clusters or breach into a pig farm. Also, we did not take into account the possibility of contaminating material being worn off during the rest of the simulated walk. In this sense, our predictions do not represent an accurate estimate of the effective number of contamination events, but rather provide an order of magnitude of the overall risk process, and a comparison of the relative risk associated with each of the different forest activities.

When focusing on this latter aspect, ASF contamination risk was generally higher for those activities, such as wild boar supplemental feeding and forest logging, which involve a repeated movement concentrated in very small areas. On the contrary, risk was relatively lower for those activities generating long wandering trajectories in bigger areas, such as wild boar hunting and individual recreational forest use. For wild boar feeding and forest logging, contamination risk was mainly affected by the probability that the small patch in which these activities occurred overlapped with the home range of an infected wild boar. Once this condition was satisfied, the specific movement patterns implied that one or more persons moved repeatedly in a very small area, eventually covering most of the available stepping surface, thus generating the relatively high ASF contamination probabilities. On the other hand, activities such as recreational forest use had a higher chance of crossing the home range of an ASF infected wild boar, but their moving trajectories did not focus specifically on those areas, causing, overall, a lower ASF contamination risk. It should be noted, though, that the actual, long-term contamination risk associated with any of the simulated forest activities was highly dependent on the frequency and intensity of the specific activity emerging from the sensitivity analysis. Therefore, although in our simulated scenarios some activities emerged as more prone to causing contamination than others, the actual evaluation of this type of risk in the real world should be done in the light of the actual field conditions. The specific frequencies and intensities of all the potentially risky human interventions in the forest, which can vary extensively depending on the local context, should be considered.

The one effect, which emerged as the most constant and significant across most of the simulated scenarios, was that related to the practice of wild boar supplemental feeding. Providing additional feed to wild boar, with the associated periodic visits needed to check and refurbish the feeding stations, was not only the riskiest activity per se (Table 2), but it also increased contamination risk for most of the other types of forest use (Fig. 3a, b). The likelihood for an individual forest user to step on a wild boar dropping was about double in an area in which wild boar were provided with supplemental feed than in one in which the practice was avoided, all the rest being equal (Table 2). The same statement is valid for most of the other types of forest use, with the only exception of forest logging, where the contamination risk was slightly lower with than without supplemental feeding, probably due to the clustered distribution of infected droppings around feeding sites and to the very small size of the simulated logging plots. The strong and generalized effect of supplemental feeding was probably due to the fact that, differently from the other simulated activities, supplemental feeding has the potential to modify wild boar space use, and the resulting spatial distribution of the infected droppings, creating clusters of highly localized infected material. The presence of feeding stations has been shown to reduce the mobility of wild boars, which tend to restrict their home range to a radius of about 1000–1500 m [28] from the artificial feeding point. In fact, they live for a large part of the year near the feeding points. Throughout the winter, wild boar movements appear to be limited to and from the feeding to the resting sites [29]. While previous epidemiological assessments have already highlighted the need to reduce wild boar supplemental feeding in ASF affected areas, because of its potential to increase wild boar density [30], our study provides the first formal evidence that this practice can also generate hotspots of potential ASF contamination, while increasing the overall risk that humans will act as unintentional vectors of the ASF virus towards new, unaffected areas, and towards pig farms. This evidence should be taken into consideration, when planning wild boar population management in ASF areas and when assembling the panel of biosecurity measures aimed at reducing the further spread of the disease.

Among the wild boar population management actions, hunting is by far the most common and widespread, for its recreational and economic value, and for its action as a population limitation tool. Moreover, hunting (followed by wild boar antigenic and serological testing) is the standard surveillance method in ASF endemic areas [22], where carcasses are difficult to detect. Several previous studies and assessments have already discussed the ASF contamination risks associated with wild boar hunting, especially when strict biosecurity measures are not implemented [14, 21, 31]. Accordingly, the third meeting of the Standing Group of African Swine Fever Experts (SGE), under the GF TADs initiative (Global Framework for Transboundary Animal Diseases), indicated that wild boar population reduction should be considered, in combination with other control measures, within the framework of a wild boar management strategy aimed at reducing ASF virus contamination of the environment [32]. In this context, our study highlights that not only wild boar carcasses and body parts, but the environment itself could be a potential source of ASF contamination during hunting. Moreover, the two types of simulated hunting techniques (collective hunting and individual hunters with dogs) exhibited similar overall contamination probabilities (Fig. 3a, b), suggesting that no hunting method can be preferred or promoted to reduce contamination risks. If we further consider that wild boar hunting and wild boar supplemental feeding are often two highly correlated management activities performed by the same people as parts of a unique population management system [33], the actual contamination risk may be higher than predicted by our simulated modelling scenarios in which the two activities were kept as independent processes.

Overall, our study highlights that there is a clear trade-off between the benefits and the risks associated with wild boar hunting in ASF affected areas. On one hand, wild boar hunting is one of the management tools for ASF control and eradication including surveillance when at low wild boar density and low virus prevalence [34]. Moreover, hunting limits wild boar densities and reduces infection rates, although it is often unable to control wild boar populations, nor achieve ASF eradication in endemic areas [35]. On the other hand, hunting generates a repeated and intense use of wild boar habitat by many persons (about 8 million in 16 European countries in 2010; [28]), with a strong spatial correlation between hunters’ movements and wild boar space use. The associated contamination risks, irrespective of all the adopted biosecurity measures, cannot be disregarded when trying to enforce an effective barrier between the wild and domestic portions of the ASF cycle.

## Conclusions

Overall, our study highlights that humans can play a relevant, although unintentional, role in the spread of the ASF virus, when using forest areas with wild boars infected with African Swine Fever. This role should be better estimated and quantified at the scale of each local context, and taken into consideration when planning wild boar management, or more generally forest management in ASF affected areas. Our study provides some general tools to quantify an order of magnitude for the number of expected contamination events, under a set of ecological and human-related parameters.

We suggest strongly limiting or avoiding wild boar supplemental feeding in ASF affected areas, as such practices generates high contamination risk and causes a spatial concentration of potentially infected biological materials, with negative consequences on the ability to confine the ASF infected animals. We also suggest seriously evaluating the trade-offs associated with the use of wild boar hunting as a management tool in ASF affected areas, with the objective to assess if its benefits (ASF active surveillance and population limitation) overcome the risks of ASF environmental contamination and human-mediated spread. Additionally and considering the relevance of ASF contamination risks in forest environments, strict biosecurity measures should be defined and enforced in ASF affected areas, not only for people whose presence in the forest is related to wild boar hunting, but for all visitors and workers potentially playing a role in virus spread.

## Supplementary Information

**Additional file 1.** Probability of African Swine Fever environmental contamination, corresponding to a 1-km simulated walk in a 50 km^2^ forest in which ASF is endemic (prevalence = 2%). The contamination probabilities are expressed in percentage and are provided for five types of forest use, at different wild boar densities and in different seasons, with (b) and without (a) wild boar artificial feeding.

**Additional file 2.** Probability of African Swine Fever environmental contamination, corresponding to five different types of forest use, simulated over a 30-day period in a 50 km^2^ forest in which ASF is endemic (prevalence = 2%). The contamination probabilities are expressed in percentage and are provided at different wild boar densities and in different seasons, with (b) and without (a) wild boar artificial feeding.

**Additional file 3.** R script for the simulation of ASF contamination risk by a single forest user.

**Additional file 4.** R script for the simulation of ASF contamination risk during forestry activities.

**Additional file 5.** R script for the simulation of ASF contamination risk during wild boar collective hunting.

**Additional file 6.** R script for the simulation of ASF contamination risk during wild boar individual hunting.

## Data Availability

The datasets used and/or analysed during the current study are available from the corresponding author on reasonable request.

## References

[1] Blome S, Gabriel C, Beer M (2013). Pathogenesis of African swine fever in domestic pigs and European wild boar. Virus Res.

[2] Penrith ML (2020). Current status of African swine fever. CABI A&B.

[3] Sauter-louis C, Conraths FJ, Probst C, Blohm U, Schulz K, Sehl J (2021). African swine fever in wild boar in Europe—a review. Viruses.

[4] Pitts N, Whitnall T (2019). Impact of African swine fever on global markets. Agric Commod.

[5] Pepin KM, Golnar AJ, Abdo Z, Podgórski T (2020). Ecological drivers of African swine fever virus persistence in wild boar populations: insight for control. Ecol Evol.

[6] Gervasi V, Guberti V (2021). African swine fever endemic persistence in wild boar populations: key mechanisms explored through modelling. Transbound Emerg Dis.

[7] Probst C, Globig A, Knoll B, Conraths FJ, Depner K (2017). Behaviour of free ranging wild boar towards their dead fellows: potential implications for the transmission of African swine fever. R Soc Open Sci.

[8] Carlson J, Fischer M, Zani L, Eschbaumer M, Fuchs W, Mettenleiter T (2020). Stability of African swine fever virus in soil and options to mitigate the potential transmission risk. Pathogens.

[9] Davies K, Goatley LC, Guinat C, Netherton CL, Gubbins S, Dixon LK (2017). Survival of African swine fever virus in excretions from pigs experimentally infected with the Georgia 2007/1 isolate. Transbound Emerg Dis.

[10] Fischer M, Hühr J, Blome S, Conraths FJ, Probst C (2020). Stability of African swine fever virus in carcasses of domestic pigs and wild boar experimentally infected. Viruses.

[11] Probst C, Gethmann J, Amler S, Globig A, Knoll B, Conraths FJ (2019). The potential role of scavengers in spreading African swine fever among wild boar. Sci Rep.

[12] Mazur-Panasiuk N, Żmudzki J, Woźniakowski G (2019). African swine fever virus-persistence in different environmental conditions and the possibility of its indirect transmission. J Vet Res.

[13] Bellini S, Casadei G, de Lorenzi G, Tamba M (2021). A review of risk factors of African swine fever incursion in pig farming within the European Union scenario. Pathogenes.

[14] Guberti V, Khomenko S, Masiulis M, Kerba S (2019). African swine fever in wild boar: ecology and biosecurity.

[15] Lamberga K, Olsevskis E, Seržants M, Berzins A, Viltrop A, Depner K (2020). African swine fever in two large commercial pig farms in Latvia—estimation of the high risk period and virus spread within the farm. Vet Sci.

[16] Lamberga K, Seržants M, Olsevskis E (2018). African swine fever outbreak investigations in a large commercial pig farm in Latvia: a case report. Berl Münch Tierärztl Wochenschr.

[17] Bellini S, Rutili D, Guberti V (2016). Preventive measures aimed at minimizing the risk of African swine fever virus spread in pig farming systems. Acta Vet Scand.

[18] European Food Safety Authority (2014). Evaluation of possible mitigation measures to prevent introduction and spread of African swine fever virus through wild boar. EFSA J.

[19] R Development Core Team (2008). R: a language and environment for statistical computing.

[20] Massei G, Genov P, Staines BW, Gorman ML (1997). Factors influencing home range and activity of wild boar (*Sus scrofa*) in a Mediterranean coastal area. J Zool.

[21] Nurmoja I, Schulz K, Staubach C, Sauter-Louis C, Depner K, Conraths FJ (2017). Development of African swine fever epidemic among wild boar in Estonia-two different areas in the epidemiological focus. Sci Rep.

[22] Nielsen SS, Alvarez J, Bicout DJ, Calistri P, Depner K, Drewe JA (2021). ASF exit strategy: providing cumulative evidence of the absence of African swine fever virus circulation in wild boar populations using standard surveillance measures. EFSA J.

[23] Gallardo MC, Reoyo ADLT, Fernández-Pinero J, Iglesias I, Muñoz MJ, Arias ML (2015). African swine fever: a global view of the current challenge. Porc Health Manag.

[24] Ferretti F, Storer K, Coats J, Massei G (2015). Temporal and spatial patterns of defecation in wild boar. Wild Soc Bull.

[25] Ballesteros C, Vicente J, Carrasco-García R, Mateo R, de la Fuente J, Gortázar C (2011). Specificity and success of oral-bait delivery to Eurasian wild boar in Mediterranean woodland habitats. Eur J Wildl Res.

[26] EFSA AHAW Panel (EFSA Panel on Animal Health and Welfare). Scientific opinion on the African swine fever in wild boar. EFSA J. 2018;16:5344. 10.2903/j.efsa.2018.5344 PMC7009363 32625980

[27] Boklund A, Cay B, Depner K, Földi Z, Guberti V, Masiulis M (2018). Epidemiological analyses of African swine fever in the European Union (November 2017 until November 2018). EFSA J.

[28] Snow NP, VerCauteren KC (2019). Movement responses inform inform effectiveness and consequences of baiting wild pigs for population control. Eur J Wildl Res.

[29] Jazek M, Kusta T, Cerveny J. Effect of supplementary feeding on spatial activity of wild boar during the winter season. In: 31st IUGB Congress, Brussels, Belgium; 2013.

[30] EFSA AHAW Panel (EFSA Panel on Animal Health and Welfare. EFSA. Scientific ppinion on African swine fever. EFSA J. 2015;13:4163.

[31] Chenais E, Depner K, Guberti V, Dietze K, Viltrop A, Ståhl K (2019). Epidemiological considerations on African swine fever in Europe 2014–2018. Porc Health Manag.

[32] GF-TADs. Standing group of experts on African swine fever in the Baltic and Eastern Europe region under the GF-TADs umbrella (Third meeting final report).

[33] Pokorny B, Ozoliņš J, Šprem N, Massei G, Kindberg J, Licoppe A (2015). Wild boar populations up, numbers of hunters down? A review of trends and implications for Europe. Pest Manag Sci.

[34] Gervasi V, Marcon A, Bellini S, Guberti V (2020). Evaluation of the efficiency of active and passive surveillance in the detection of African swine fever in wild boar. Vet Sci.

[35] Gervasi V, Guberti V (2022). Combining hunting and intensive carcass removal to eradicate African swine fever from wild boar populations. Prev Vet Med.

