# Supplementary material for: Estimating the risk of environmental contamination by forest users in African Swine Fever endemic areas
Source: Acta Vet Scand. 2022 Jul 27;64:16. doi: 10.1186/s13028-022-00636-z (PMC9327371; doi:10.1186/s13028-022-00636-z)
Supplement: Supplementary file 1 — Additional file 1. Probability of African Swine Fever environmental contamination, corresponding to a 1-km simulated walk in a 50 km2 forest in which ASF is endemic (prevalence = 2%). The contamination probabilities are expressed in percentage and are provided for five types of forest use, at different wild boar densities and in different seasons, with (b) and without (a) wild boar artificial feeding. [file 13028_2022_636_MOESM1_ESM.docx]

**Additional File 1 – Probability of African Swine Fever environmental contamination, corresponding to a 1-km simulated walk in a 50 km^2^ forest in which ASF is endemic (prevalence = 2%). The contamination probabilities are expressed in percentage and are provided for five types of forest use, at different wild boar densities and in different seasons, with (b) and without (a) wild boar artificial feeding.**

| Type of forest use | Season | Wild boar density | | | |
| --- | --- | --- | --- | --- | --- |
|  |  | 1.0 / km^2^ | | 3.0 / km^2^ | |
|  |  | Supplemental feeding | | | |
|  |  | No | Yes | No | Yes |
| Individual | Winter | 0.012% | 0.020% | 0.022% | 0.031% |
|  | Summer | 0.005% | 0.014% | 0.017% | 0.024% |
| Wild boar hunt (collective drive) | Winter | 0.009% | 0.011% | 0.023% | 0.026% |
| Wild boar hunt (single hunter with dogs) | Winter | 0.09% | 0.124% | 0.221% | 0.267% |
| Forest logging | Winter | 0.881% | 0.791% | 2.437% | 2.264% |
|  | Summer | 0.562% | 0.483% | 1.546% | 1.446% |
| Feeding site visit | Winter | - | 1.864% | - | 2.481% |
|  | Summer | - | 1.169% | - | 2.014% |
