# Supplementary material for: Estimating the risk of environmental contamination by forest users in African Swine Fever endemic areas
Source: Acta Vet Scand. 2022 Jul 27;64:16. doi: 10.1186/s13028-022-00636-z (PMC9327371; doi:10.1186/s13028-022-00636-z)
Supplement: Supplementary file 2 — Additional file 2. Probability of African Swine Fever environmental contamination, corresponding to five different types of forest use, simulated over a 30-day period in a 50 km2 forest in which ASF is endemic (prevalence = 2%). The contamination probabilities are expressed in percentage and are provided at different wild boar densities and in different seasons, with (b) and without (a) wild boar artificial feeding. [file 13028_2022_636_MOESM2_ESM.docx]

**Additional File 2 – Probability of African Swine Fever environmental contamination, corresponding to five different types of forest use, simulated over a 30-day period in a 50 km^2^ forest in which ASF is endemic (prevalence = 2%). The contamination probabilities are expressed in percentage and are provided at different wild boar densities and in different seasons, with (b) and without (a) wild boar artificial feeding.**

| Type of forest use | Season | Wild boar density | | | |
| --- | --- | --- | --- | --- | --- |
|  |  | 1.0 / km^2^ | | 3.0 / km^2^ | |
|  |  | Supplemental feeding | | | |
|  |  | No | Yes | No | Yes |
| Individual | Winter | 7.7% | 11.5% | 14.2% | 21.6% |
|  | Summer | 5.3% | 8.0% | 9.9% | 15.1% |
| Wild boar hunt (collective drive) | Winter | 10.4% | 12.7% | 25.9% | 29.7% |
| Wild boar hunt (single hunter with dogs) | Winter | 11.6% | 13.4% | 26.6% | 30.8% |
| Forest logging | Winter | 17.5% | 17.0% | 48.4% | 44.9% |
|  | Summer | 11.1% | 9.6% | 30.7% | 28.7% |
| Feeding site visit | Winter | - | 60.9% | - | 71.6% |
|  | Summer | - | 44.4% | - | 63.8% |
