# Supplementary material for: Estimating the risk of environmental contamination by forest users in African Swine Fever endemic areas
Source: Acta Vet Scand. 2022 Jul 27;64:16. doi: 10.1186/s13028-022-00636-z (PMC9327371; doi:10.1186/s13028-022-00636-z)
Supplement: Supplementary file 3 — Additional file 3. R script for the simulation of ASF contamination risk by a single forest user. [file 13028_2022_636_MOESM3_ESM.docx]

**Additional File 3** – R script for the simulation of ASF contamination risk by a single forest user.

#################

# LOAD LIBRARIES#

#################

require(spatstat)

require(maptools)

library(rgeos)

library(sp)

library(adehabitatLT)

library(spdep)

library(rgdal)

library(raster)

library(sf)

library(geosphere)

###############################

# Set simulation parameters##

###############################

niter=10000 # n. iterations

npersons=1 # n. forest users

ndays=1 # n. days of simulated forest use

results <- rep(NA,niter)

sito <- T # does the scenario include wild boar feeding sites

nscats <- 36 # number of simulated contaminated droppings

popsize <- 3 # number of ASF infected wild boar

traj_length <- 5 # length of trajectory in m

stepsize <- 1 # length of a straigth segment during which humans walk without changing direction

nsteps <- traj_length/stepsize # resulting number of turns in the trajectory

###############################

# Set parameters for the grid##

###############################

# TOTAL STUDY AREA IN SQ_KM

area <- 50

cell_size <- 1

# N CELLS IN THE STUDY AREA, RESULTING FROM THE TOTAL STUDY AREA AND THE CELL SIZE

ncells <- round(((sqrt(area))/cell_size),0)^2

lato <- sqrt(area)

##################################################

# Set parameters for the wild boar population #

##################################################

# WILD BOAR HOME RANGE SIZE IN kmsq

HRsize <- 1

# HOME RANGE RADIUS, DERIVED FROM HOME RANGE SIZE

rHR <- sqrt(HRsize/3.14)

############################################################################

####### GENERATE A SAMPLING GRID #############

############################################################################

# generate a sqaured grid based on cell size and number of cells as defined above

grd <- GridTopology(c(0,0), c(cell_size,cell_size), c(sqrt(ncells),sqrt(ncells))) # build a grid with coordinates of the first cell, the step used to create the grid (by=) and the number of cells

# transform the grid into a spatial polygon object

polys <- as.SpatialPolygons.GridTopology(grd) # transformation into a SpatialPolygon

# generate a single polygon of the same extent as the grid, which defines the whole stidy area

study_area <- owin(xrange=c(polys@polygons[1][[1]]@Polygons[[1]]@coords[1,1],polys@polygons[length(polys)][[1]]@Polygons[[1]]@coords[3,1]), yrange=c(polys@polygons[length(polys)][[1]]@Polygons[[1]]@coords[1,2],polys@polygons[1][[1]]@Polygons[[1]]@coords[2,2]))

study_area_sp <- gUnaryUnion(polys)

proj4string(polys)<-CRS("+init=epsg:32632") # Assign UTM32N CRS

proj4string(study_area_sp)<-CRS("+init=epsg:32632") # Assign UTM32N CRS

############################################################################

####### GENERATE FEEDING POINTs IN THE CENTRE OF EACH CELL ########

############################################################################

x_feed <- rep(0,length(polys))

y_feed <- rep(0,length(polys))

for (w in 1:length(polys)){

x_feed[w] <- (as.numeric(polys@polygons[w][[1]]@Polygons[[1]]@coords[1,1])+ as.numeric(polys@polygons[w][[1]]@Polygons[[1]]@coords[3,1])) /2

y_feed[w] <- (as.numeric(polys@polygons[w][[1]]@Polygons[[1]]@coords[1,2])+ as.numeric(polys@polygons[w][[1]]@Polygons[[1]]@coords[3,2])) /2

feeding_points <- SpatialPoints(cbind(x_feed, y_feed))

}

proj4string(feeding_points)<-CRS("+init=epsg:32632") # Assign UTM32N CRS

############################################################################

####### GENERATE A 200 m radius IN THE CENTRE of each feeding station ############

############################################################################

feeding_area <- gBuffer(feeding_points[1], byid=T, width=0.2)

for (w in 2:length(polys)){

new_area <-gBuffer(feeding_points[w], byid=T, width=0.2) # a buffer around the centers to have a disk with the radius rHR

row.names(new_area) <- as.character(w)

feeding_area <- spRbind(feeding_area, new_area)

}

proj4string(feeding_area)<-CRS("+init=epsg:32632") # Assign UTM32N CRS

for (k in 1:niter){

#############################################################################################

####### GENERATE A RANDOM POINT PROCESS TO SIMULATE HOME RANGE CENTERS #########

#############################################################################################

# generate a random point pattern

HR <- rpoint(popsize, fmax=NULL, win=study_area)

# transformation of the HR centers into Spatial Points

HRsp <- as(HR, "SpatialPoints")

# creates a buffer around each home range centre, based on the home range radius defined above

HR2sp <- gBuffer(HRsp, byid=T, width=rHR) # a buffer around the centers to have a disk with the radius rHR

proj4string(HR2sp)<-CRS("+init=epsg:32632") # Assign UTM32N CRS

############################################################

# IDENTIFY FEEDING AND NON FEEDING AREAS IN EACAH HOME RANGE#

#############################################################

if (sito ==T){

HR_feed <- intersect(HR2sp[1], feeding_area)

HR_nofeed <- gDifference(HR2sp[1], HR_feed)

nscats1 <- round(nscats*0.66,0) # define the total number of scats left by each wild boar

nscats2 <- round(nscats*0.33,0)

}

if (sito ==F){

HR_feed <- HR2sp[1]

HR_nofeed <- HR2sp[1]

nscats1 <- round(nscats*0.5,0) # define the total number of scats left by each wild boar

nscats2 <- round(nscats*0.5,0)

}

##############################################################################################

####### GENERATE AN AGGREGATED POINT PROCESS TO SIMULATE WILD BOAR SCATS #########

#############################################################################################

# generate scats within each home range and then merge them into a single object

scats_sp1 = SpatialPoints(data.frame(x = 0, y = 0))[-1,]

scats1 <- rpoint(nscats1, fmax=NULL, win=HR_feed)

scats_sp1 <- rbind(scats_sp1, as(scats1, "SpatialPoints"))

scats_sp2 = SpatialPoints(data.frame(x = 0, y = 0))[-1,]

scats2 <- rpoint(nscats2, fmax=NULL, win=HR_nofeed)

scats_sp2 <- rbind(scats_sp2, as(scats2, "SpatialPoints"))

if (popsize>1){

for (q in 2:popsize){

if (sito ==T){

HR_feed <- intersect(HR2sp[q], feeding_area)

HR_nofeed <- gDifference(HR2sp[q], HR_feed)

nscats1 <- round(nscats*0.66,0) # define the total number of scats left by each wild boar

nscats2 <- round(nscats*0.33,0)

}

if (sito ==F){

HR_feed <- HR2sp[q]

HR_nofeed <- HR2sp[q]

nscats1 <- round(nscats*0.5,0) # define the total number of scats left by each wild boar

nscats2 <- round(nscats*0.5,0)

}

# generate scats within each home range and then merge them into a single object

scats1 <- rpoint(nscats1, fmax=NULL, win=HR_feed)

scats_sp1 <- rbind(scats_sp1, as(scats1, "SpatialPoints"))

scats2 <- rpoint(nscats2, fmax=NULL, win=HR_nofeed)

scats_sp2 <- rbind(scats_sp2, as(scats2, "SpatialPoints"))

}

}

proj4string(scats_sp1)<-CRS(proj4string(polys))

proj4string(scats_sp2)<-CRS(proj4string(polys))

############################################################################

####### Simulating human movement process ############

############################################################################

ntracks <- ndays*npersons

track_results <- rep(0,ntracks)

for (i in 1:ntracks){

#GENERATE A STARTING POINT

traj_length <- 10 # length of trajectory in m

stepsize <- 1 # length of a straigth segment during which humans walk without changing direction

nsteps <- traj_length/stepsize # resulting number of turns in the trajectory

# generate a random starting point

traj <- t(matrix(c(runif(1,min=extent(polys)[1], max=extent(polys)[2]),

runif(1,min=extent(polys)[3], max=extent(polys)[4]))))

# generate successive steps of fixed length, given by stepsize, and with a random angle between 0 and 360.

# Angles which lead outside the study area are removed,

# so that the trajectory always stays within the study area.

for (z in 2:nsteps){

t <- seq(0,2*pi,length=360) # Create a wide pool of angles (n=360)

buff <- t(rbind(traj[nrow(traj),1]+sin(t)*stepsize, traj[nrow(traj),2]+cos(t)*stepsize)) # Create a pool of new points amongst which to choose the the next step, starting from the last point (i.e. traj[nrow(traj),])

buff_sp <- SpatialPoints(buff, proj4string = CRS(proj4string(polys)))

overlay <- over(buff_sp, polys) # Overlay the potential new steps and the study area

angle <- sample(which(!is.na(overlay)),size=1) # Randomly choose one of the new steps that are inside the study area

traj <- rbind(traj, buff[angle,]) # Append the selected new step to our data set

}

#plot what has been generated so far

traj_points <- SpatialPoints(traj, proj4string = CRS(proj4string(polys)))

traj_sp <- SpatialLines(list(Lines(list(Line(traj)), "id")),

proj4string = CRS(proj4string(polys)))

plot(study_area_sp, main = "")

plot(polys,add=T) # Here "polys" is the same as "study_area"

plot(feeding_area, add=T, col="darkolivegreen1")

plot(HR2sp, add=T, col="brown1")

plot(feeding_points, add=T)

plot(scats_sp1, add=T, col="black", pch=20)

plot(scats_sp2, add=T, col="black", pch=20)

plot(traj_sp, add=T, col="black", lwd=2)

############################################################################

####### Measure distance between trajectory and scats ###

############################################################################

# it gives a warning regarding the projection, but in our case it makes no difference because the study area is small

# Convert scats and tracks into LAT/LONG (function dist2line requires so)

scats_sp1_WGS84<-spTransform(scats_sp1, CRSobj = CRS("+init=epsg:4326"))

scats_sp2_WGS84<-spTransform(scats_sp2, CRSobj = CRS("+init=epsg:4326"))

traj_sp_WGS84<-spTransform(traj_sp, CRSobj = CRS("+init=epsg:4326"))

# Test for distance calculation

traj_scat1_dist2 <- dist2Line(scats_sp1_WGS84, traj_sp_WGS84, distfun=distGeo)[,1]

traj_scat2_dist2 <- dist2Line(scats_sp2_WGS84, traj_sp_WGS84, distfun=distGeo)[,1]

# test if any point on the trajectory is closer than one meter to any scat

detected1 <- sum(traj_scat1_dist2>0 & traj_scat1_dist2 < 0.0002)

detected2 <- sum(traj_scat2_dist2>0 & traj_scat2_dist2 < 0.0002)

track_results[i] <- sum(detected1, detected2)

}

results[k] <- sum(track_results)

}

# account for the fact that only 1/3 of the trajectory is made of steps

results2 <- rep(0, niter)

for (i in 1:length(results2)){

if (results[i]>0){ results2[i] <- as.numeric(sum(rbinom(n=results[i],size=1,prob = 0.33))>0)

}

}

#probability to bring infection away

p <- ((sum(results2>0) / (100/niter)))

p
