# Supplementary material for: Estimating the risk of environmental contamination by forest users in African Swine Fever endemic areas
Source: Acta Vet Scand. 2022 Jul 27;64:16. doi: 10.1186/s13028-022-00636-z (PMC9327371; doi:10.1186/s13028-022-00636-z)
Supplement: Supplementary file 6 — Additional file 6. R script for the simulation of ASF contamination risk during wild boar individual hunting. [file 13028_2022_636_MOESM6_ESM.docx]

**Additional File 6 – R script for the simulation of ASF contamination risk during wild boar individual hunting.**

#################

# LOAD LIBRARIES#

#################

require(spatstat)

require(maptools)

library(rgeos)

library(sp)

library(unmarked)

library(adehabitatLT)

library(spdep)

library(rgdal)

library(raster)

library(sf)

library(geosphere)

library(sde)

###############################

# Set simulation parameters##

###############################

niter=100

npersons=1

ndays=1

results <- rep(0,niter)

walked <- rep(0,niter)

sito <- F

nscats <- 36

popsize <- 50

n.infected <- 1

traj_length <- 5 # length of trajectory in m

stepsize <- 1 # length of a straigth segment during which humans walk without changing direction

nsteps <- traj_length/stepsize # resulting number of turns in the trajectory

###############################

# Set parameters for the grid##

###############################

# TOTAL STUDY AREA IN SQ_KM

area <- 50

cell_size <- 1

# N CELLS IN THE STUDY AREA, RESULTING FROM THE TOTAL STUDY AREA AND THE CELL SIZE

ncells <- round(((sqrt(area))/cell_size),0)^2 #arrotonda la 2 dec

lato <- sqrt(area)

##################################################

# Set parameters for the wild boar population #

##################################################

# WILD BOAR HOME RANGE SIZE IN M2 1 kmsq in one night (Spitz and janeau 1990); 4 kmsq in one month (Boitani et al. 1994)

HRsize <- 1

# HOME RANGE RADIUS, DERIVED FROM HOME RANGE SIZE

rHR <- sqrt(HRsize/3.14)

############################################################################

####### GENERATE A SAMPLING GRID #############

############################################################################

# generate a sqaured grid based on cell size and number of cells as defined above

grd <- GridTopology(c(0,0), c(cell_size,cell_size), c(sqrt(ncells),sqrt(ncells))) # build a grid with coordinates of the first cell, the step used to create the grid (by=) and the number of cells

# transform the grid into a spatial polygon object

polys <- as.SpatialPolygons.GridTopology(grd) # transformation into a SpatialPolygon

# generate a single polygon of the same extent as the grid, which defines the whole stidy area

study_area <- owin(xrange=c(polys@polygons[1][[1]]@Polygons[[1]]@coords[1,1],polys@polygons[length(polys)][[1]]@Polygons[[1]]@coords[3,1]), yrange=c(polys@polygons[length(polys)][[1]]@Polygons[[1]]@coords[1,2],polys@polygons[1][[1]]@Polygons[[1]]@coords[2,2]))

study_area_sp <- gUnaryUnion(polys)

proj4string(polys)<-CRS("+init=epsg:32632") # Assign UTM32N CRS

proj4string(study_area_sp)<-CRS("+init=epsg:32632") # Assign UTM32N CRS

############################################################################

####### GENERATE FEEDING POINTs IN THE CENTRE OF EACH CELL ########

############################################################################

x_feed <- rep(0,length(polys))

y_feed <- rep(0,length(polys))

for (w in 1:length(polys)){

x_feed[w] <- (as.numeric(polys@polygons[w][[1]]@Polygons[[1]]@coords[1,1])+ as.numeric(polys@polygons[w][[1]]@Polygons[[1]]@coords[3,1])) /2

y_feed[w] <- (as.numeric(polys@polygons[w][[1]]@Polygons[[1]]@coords[1,2])+ as.numeric(polys@polygons[w][[1]]@Polygons[[1]]@coords[3,2])) /2

feeding_points <- SpatialPoints(cbind(x_feed, y_feed))

}

proj4string(feeding_points)<-CRS("+init=epsg:32632") # Assign UTM32N CRS

############################################################################

####### GENERATE A 200 m radius IN THE CENTRE of each feeding station ############

############################################################################

feeding_area <- gBuffer(feeding_points[1], byid=T, width=0.2)

for (w in 2:length(polys)){

new_area <-gBuffer(feeding_points[w], byid=T, width=0.2) # a buffer around the centers to have a disk with the radius rHR

row.names(new_area) <- as.character(w)

feeding_area <- spRbind(feeding_area, new_area)

}

proj4string(feeding_area)<-CRS("+init=epsg:32632") # Assign UTM32N CRS

for (k in 1:niter){

#############################################################################################

####### GENERATE A RANDOM POINT PROCESS TO SIMULATE HOME RANGE CENTERS #########

#############################################################################################

# generate a random point pattern

HR <- rpoint(popsize, fmax=NULL, win=study_area)

######################################################################

### SHOULD WE MAKE SURE THAT THE WHOLE HR IS INSIDE OUR STUDY AREA? ###

### i.e. THE RANDOM POINTS SHOULD BE AT LEAST AT rHR DISTANCE FROM THE BORDER OF THE STUDY AREA ###

######################################################################

# transformation of the HR centers into Spatial Points

HRsp <- as(HR, "SpatialPoints")

# creates a buffer around each home range centre, based on the home range radius defined above

HR2sp <- gBuffer(HRsp, byid=T, width=rHR) # a buffer around the centers to have a disk with the radius rHR

proj4string(HR2sp)<-CRS("+init=epsg:32632") # Assign UTM32N CRS

HR2isp <- HR2sp[1:n.infected,]

############################################################

# IDENTIFY FEEDING AND NON FEEDING AREAS IN EACAH HOME RANGE#

#############################################################

if (sito ==T){

HR_feed <- intersect(HR2isp, feeding_area)

HR_nofeed <- gDifference(HR2isp, HR_feed)

nscats1 <- round(nscats*0.66,0)*n.infected # define the total number of scats left by each wild boar

nscats2 <- round(nscats*0.33,0)*n.infected

}

if (sito ==F){

HR_feed <- HR2isp

HR_nofeed <- HR2isp

nscats1 <- round(nscats*0.5,0)*n.infected # define the total number of scats left by each wild boar

nscats2 <- round(nscats*0.5,0)*n.infected

}

##############################################################################################

####### GENERATE AN AGGREGATED POINT PROCESS TO SIMULATE WILD BOAR SCATS #########

#############################################################################################

# generate scats within each home range and then merge them into a single object

scats_sp1 = SpatialPoints(data.frame(x = 0, y = 0))[-1,]

scats1 <- rpoint(nscats1, fmax=NULL, win=HR_feed)

scats_sp1 <- rbind(scats_sp1, as(scats1, "SpatialPoints"))

scats_sp2 = SpatialPoints(data.frame(x = 0, y = 0))[-1,]

scats2 <- rpoint(nscats2, fmax=NULL, win=HR_nofeed)

scats_sp2 <- rbind(scats_sp2, as(scats2, "SpatialPoints"))

if (sito ==F){

HR_feed <- HR2isp

HR_nofeed <- HR2isp

nscats1 <- round(nscats*0.5,0)*n.infected # define the total number of scats left by each wild boar

nscats2 <- round(nscats*0.5,0)*n.infected

}

# generate scats within each home range and then merge them into a single object

scats1 <- rpoint(nscats1, fmax=NULL, win=HR_feed)

scats_sp1 <- rbind(scats_sp1, as(scats1, "SpatialPoints"))

scats2 <- rpoint(nscats2, fmax=NULL, win=HR_nofeed)

scats_sp2 <- rbind(scats_sp2, as(scats2, "SpatialPoints"))

proj4string(scats_sp1)<-CRS(proj4string(polys))

proj4string(scats_sp2)<-CRS(proj4string(polys))

############################################################################

####### Simulating human movement process ############

############################################################################

ntracks <- ndays*npersons

track_results <- rep(0,ntracks)

hunted <- 1

#round(runif(1, min=1, max=popsize))

if (hunted <= n.infected){

hunted_xy <- matrix(c(coordinates(HR2sp)[hunted,1], coordinates(HR2sp)[hunted,2]), nrow=1, ncol=2)

colnames(hunted_xy) <- c("x", "y")

hunted_sp <- SpatialPoints(hunted_xy)

for (i in 1:ntracks){

#GENERATE A STARTING POINT

traj_length <- 100 # length of trajectory in m

traj <- simm.mou(date = c(1:traj_length), x0=as.numeric(c(coordinates(HR2sp)[hunted,1] + rnorm(1, mean= 0, sd = 0.4), coordinates(HR2sp)[hunted,2]+ rnorm(1, mean= 0, sd = 0.4))),

b=as.numeric(c(coordinates(HR2sp)[hunted,1], coordinates(HR2sp)[hunted,2])), a = diag(c(0.1, 0.1)), sigma = diag(c(0.1,0.1)))

traj_sp <- SpatialPoints(cbind(traj[[1]]$x, traj[[1]]$y)) # transformation of the HR centers into Spatial Points

traj_line <- as(traj_sp,"SpatialLines")

proj4string(traj_line)<-CRS(proj4string(polys))

plot(study_area_sp, main = "")

plot(polys,add=T) # Here "polys" is the same as "study_area"

plot(feeding_area, add=T, col="darkolivegreen1")

plot(HR2sp, add=T)

plot(HR2isp, add=T, col="brown1")

plot(feeding_points, add=T)

plot(scats_sp1, add=T, col="black", pch=20)

plot(scats_sp2, add=T, col="black", pch=20)

plot(traj_line, add=T, lwd=2)

plot(HR2sp[hunted,], add=T)

walked[k] <- gLength(traj_line)

############################################################################

####### Measure distance between trajectory and scats ###

############################################################################

# it gives a warning regarding the projection, but in our case it makes no difference because the study area is small

# Convert scats and tracks into LAT/LONG (function dist2line requires so)

scats_sp1_WGS84<-spTransform(scats_sp1, CRSobj = CRS("+init=epsg:4326"))

scats_sp2_WGS84<-spTransform(scats_sp2, CRSobj = CRS("+init=epsg:4326"))

traj_sp_WGS84<-spTransform(traj_line, CRSobj = CRS("+init=epsg:4326"))

# Test for distance calculation

traj_scat1_dist2 <- dist2Line(scats_sp1_WGS84, traj_sp_WGS84, distfun=distGeo)[,1]

traj_scat2_dist2 <- dist2Line(scats_sp2_WGS84, traj_sp_WGS84, distfun=distGeo)[,1]

# test if any point on the trajectory is closer than one meter to any scat

detected1 <- sum(traj_scat1_dist2>0 & traj_scat1_dist2 < 0.0002)

detected2 <- sum(traj_scat2_dist2>0 & traj_scat2_dist2 < 0.0002)

track_results[i] <- sum(detected1, detected2)

}

results[k] <- sum(track_results)

}

}

# account for the fact that only 1/3 of the trajectory is made of steps

results2 <- rep(0, niter)

for (i in 1:length(results2)){

if (results[i]>0){ results2[i] <- as.numeric(sum(rbinom(n=results[i],size=1,prob = 0.33))>0)

}

}

walked_tot <- sum(walked[results2>0])

#probability to bring infection away

((sum(results2>0) / niter) *100)

walked_tot

p <- ((sum(results2>0) / (100/niter)))

p
